# A Functional Analysis of the Cyclophilin Repertoire in the Protozoan Parasite *Trypanosoma Cruzi*

**DOI:** 10.3390/biom8040132

**Published:** 2018-10-31

**Authors:** Alina E. Perrone, Natalia Milduberger, Alicia G. Fuchs, Patricia L. Bustos, Jacqueline Bua

**Affiliations:** 1 Instituto Nacional de Parasitología “Dr. Mario Fatala Chaben”—ANLIS C.G. Malbrán, Paseo Colón 568-C1282AFF Buenos Aires, Argentina; alinaperrone@yahoo.com.ar (A.E.P.); nmilduberger@gmail.com (N.M.); alicia.fuchs@uai.edu.ar (A.G.F.); pato54mar@yahoo.com.ar (P.L.B.); 2 CAECIHS, Universidad Abierta Interamericana, Av. Montes de Oca 745, 2º piso, C1270AAH Buenos Aires, Argentina

**Keywords:** *Trypanosoma cruzi*, Chagas disease, protozoan parasite, cyclosporin A, non-immunosuppressive analogs, trypanocidal compounds, cell death, cyclophilins, *Tc*CyP19, *Tc*CyP22

## Abstract

*Trypanosoma cruzi* is the etiological agent of Chagas disease. It affects eight million people worldwide and can be spread by several routes, such as vectorborne transmission in endemic areas and congenitally, and is also important in non-endemic regions such as the United States and Europe due to migration from Latin America. Cyclophilins (CyPs) are proteins with enzymatic peptidyl-prolyl isomerase activity (PPIase), essential for protein folding in vivo. Cyclosporin A (CsA) has a high binding affinity for CyPs and inhibits their PPIase activity. CsA has proved to be a parasiticidal drug on some protozoa, including *T. cruzi*. In this review, we describe the *T. cruzi* cyclophilin gene family, that comprises 15 paralogues. Among the proteins isolated by CsA-affinity chromatography, we found orthologues of mammalian CyPs. *Tc*CyP19, as the human CyPA, is secreted to the extracellular environment by all parasite stages and could be part of a complex interplay involving the parasite and the host cell. *Tc*CyP22, an orthologue of mitochondrial CyPD, is involved in the regulation of parasite cell death. Our findings on *T. cruzi* cyclophilins will allow further characterization of these processes, leading to new insights into the biology, the evolution of metabolic pathways, and novel targets for anti-*T. cruzi* control.

## 1. Introduction to *Trypanosoma cruzi* Infection and Chagas Disease

*Trypanosoma cruzi* is an hemoflagellate parasitic protozoon and the etiological agent of Chagas disease. This parasitosis has epidemiological relevance affecting eight million people, mainly in South America. It is a public health priority in endemic areas because in the long term, 30% of *T. cruzi* chronic infected humans will develop serious and irreversible complications due by parasite invasion of muscles and autonomic peripheral nervous system. There are 30,000 new reported cases of Chagas each year in the Americas and 14,000 people die as a result of the disease, while more than 70 million people live in areas where there is high-risk of transmission [1]. This Kinetoplastid unicellular parasite circulates between mammalian hosts and insect vectors, which include a variety of species of Reduviidae (blood-sucking insects also known as kissing bugs), widely distributed from Southern United States to Argentina [2,3]. *T. cruzi* can be transmitted through other routes than vectorial spread, as mother to child transmission during pregnancy, and with less epidemiological impact, the parasite can be transmitted through blood transfusions, organ transplants, and oral routes [4].

Politics in public health to control Chagas disease were successful, considering that the vector transmission of *T. cruzi* has been interrupted in 17 out of 21 affected countries in the Americas. Additionally blood to be transfused is almost universally screened in blood banks [5]. Now, the main challenge is to maintain the parasite control achievements so far and prevent an actual main route of transmission: the congenital Chagas disease. A baby born to a mother infected with *T. cruzi* has between 2–10% chance of contracting this infection during gestation and birth [6,7].

The maternal-child *T. cruzi* infection has a growing importance in endemic area, since around 1.1 million women of child-bearing age in the region are infected with the *T. cruzi* parasite, and almost 9000 babies are born each year infected with *T. cruzi*, accounting for 30% of all new infections [1,7]. The non-vectorial *T. cruzi* transmission also has a conspicuous role in non-endemic countries, in which millions of Latin American immigrants are hosted. The *T. cruzi* infection has been increasingly detected in USA, with more than 300,000 people infected [8], and in Canada, the Western Pacific countries and Europe, where more than 60,000 infected people have been detected in Spain alone [9,10].

Currently, only benznidazole and nifurtimox are recognized by the World Health Organization as effective drugs for treatment of Chagas disease. Benznidazole produces a clear trypanocidal effect in humans and plays an essential role in primary and secondary prevention. Because of the challenges involved in confirming a cure for Chagas disease, benznidazole’s benefit is more readily demonstrated during the acute phase. Every effort should be made to identify and treat patients early, before Chagas disease progresses to an advanced chronic form, especially women of gestational age, considering that when *T. cruzi*-infected women are treated with benznidazole, congenital transmission is prevented in subsequent births, with a tremendous public health impact since congenital infection is a major transmission route [11].

## 2. The *T. cruzi* Cyclophilin Repertoire

*Trypanosoma cruzi*, as most of the organisms studied to date, has a family of ubiquitous and highly conserved proteins named cyclophilins that mediate protein folding events, catalizing the interconversion of the *cis* and *trans* isomers of peptidyl-prolyl bonds in peptides and proteins (peptidyl-prolyl *cis*-*trans* isomerase (PPIase) activity) [12], an enzymatic activity inhibited by the immunosuppressive undecapeptide Cyclosporin A (CsA) [13]. This cyclic peptide has a conformational polymorphism, given by a dependence of its structure and dynamics on the solution environment, that allows it to more readily move through a lipid bilayer and ensures its membrane permeability, increasing its therapeutic potential [14].

The *T. cruzi* cyclophilin of 19 kDa, *Tc*CyP19, has a 72% protein sequence identity with the human CyPA, and shows a high sequence identity with other Trypanosomatids of sanitary importance, as *Trypanosoma brucei* (90%) and *Leishmania* spp. (79–81%) [15]. Using the protein sequence of the archetypal cyclophilin *Tc*CyP19 we have searched sequence identities through the Blast Program [16] in the available databases, and found 15 unique *T. cruzi* cyclophilin paralogues ranging from 19–110 kDa, some of them with predicted subcellular localization signals. Bioinformatic analysis established that each *Tc*CyP has a unique cyclophilin-like domain, which displays the crucial amino acid residues for PPIase activity, and most of the components of this parasite CyP family show the tryptophan residue essential for CsA binding [17].

Seven *T. cruzi* cyclophilin gene family members are transcribed to mRNA: *Tc*CyPs of 19, 20, 21, 22, 25, 28, and 40 kDa. DNA hybridizations performed with labelled cDNAs revealed these CyPs were organized as a single copy gene in the *T. cruzi* DNA haploid genome and bound to two different chromosomal bands. Only five *T. cruzi* cyclophilins were found as expressed proteins: *Tc*CyP19, *Tc*CyP22, *Tc*CyP28 and *Tc*CyP40 were isolated by CsA-affinity chromatography [17] and one cyclophilin of 21 kDa (*Tc*CyP21) could only be detected by immunoblotting assays on a membrane-enriched fraction, as it was found to be a low-abundance protein [18]. Orthologues to the human cyclophilin hCyP40 [19], found in Hsp90-containing protein complexes, have also been found in the genomes of *T. cruzi*, and *Leishmania* spp. This type of cyclophilin contains two signature motifs, the C-terminal three tetratricopeptide repeats (TPRs) [20], involved in protein-protein interaction and the N-terminal cyclophilin-like domain. The TPRs hydrophobic motif are the interaction surfaces necessary for binding to Hsp90 [21]. It was reported that human CyP-40 had a weak affinity CsA since a histidine (H) residue replaced a tryptophan (W), critical for its binding. The W is highly conserved in other cyclophilins with high affinity for CsA. However, parasitic protozoan cyclophilins of 40 kDa *Tc*CyP40 and *Leishmania* CyP40 could be isolated by CsA affinity chromatography [17,22].

**Figure 1:**
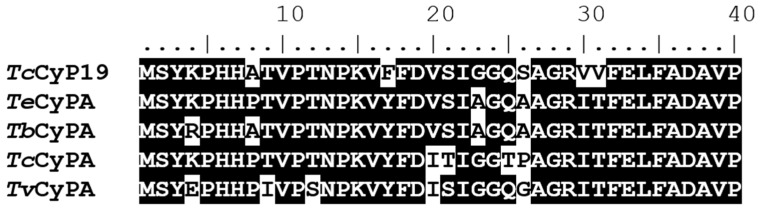
Secreted Kinetoplastid cyclophilins protein sequences. The first 40 amino acid residues of *T. cruzi* cyclophilin 19 (Acc No. AF191832), were aligned with the protein sequences of *T. evansi* (*Te*CyPA; Acc No. ABI20435), *T. brucei* CyPA (*Tb*CyPA; Acc No. U68270), *T. congolense* (*Tc*CyPA; Acc No. U68268), *T. vivax* CyPA (*Tv*CyPA; Acc No. U68269). Identical amino acids respect to the *Tc*CyP19 protein sequence are shadowed.

The secretion of cytosolic cyclophilins to the extracellular space has been described in human cells, and other organisms [23]. Parasite orthologues of hCyPA have also been localized to cytosol and secreted to the surrounding enviroment, like *Tb*CyP19 in *Trypanosoma brucei*, *T. evansi*, *T. congolense*, *T. vivax* [24], *Tg*CyP19 in *Toxoplasma gondii* [25], and *Tc*CyP19 in *T. cruzi* [26]. These trypanosomatid cytosolic cyclophilins present two consensus sequences: Asn-X-Thr and Asn-X-Ser in which the asparagine (Asn 81 and 121) or the serine/threonine (Thr 83 and Ser 123) residues of these motifs could be *N*-linked or *O*-linked glycosylated, where they would contribute to their transit through the endoplasmic reticulum (ER) [24]. However, in the first 40 residues of hCyPA orthologues in kinetoplastids, compared in Figure 1, no classical signal peptide could be predicted by internet resources as iPsort, wolfpsort, SecretomeP 2.0 [27], nor Signal P. It is known that secretory proteins not transported through the classical ER-Golgi secretory pathway are secreted through unconventional mechanisms in the absence of an ER signal sequence, such as lysine acetylation or other posttranslational modifications [28].

## 3. Cyclosporin A as Trypanocidal Agent

Since the immunosuppressive drug CsA and cyclosporin derivatives with reduced or non-immunosuppressive properties were reported as parasiticidal against *Leishmania tropica* [29], *Schistosoma mansoni* [30] and *Plasmodium falciparum* [31], parasite cyclophilins received attention as attractive potential drug targets. The anti-parasitic activity of CsA has been demonstrated in numerous protozoan and helminth parasites, reviewed in [32,33,34,35]. CsA has demonstrated a similar micromolar range of parasiticidal activity on *T. cruzi* parasites (IC_50_ = 5.9 µM) [36] compared to benznidazole (IC_50_ = 10.0 µM) for CL Brener epimastigotes [37] and Nifurtimox (Colonia, Germany) (IC_50_ = 3–5 µM) on several reference strains and isolates [38].

However, in experimental mice models the effects of CsA showed an exacerbation of the *T. cruzi* infection due to its immunosuppressive properties [39]. Since CsA could not be considered a trypanocidal drug in vivo, we searched synthetic CsA modified compounds, which were non-immunosuppressive CsA analogs, to test if they exerted any trypanocidal activity. We got several compounds from the group of CsA analogs with non-immunosupressive activity available, through generous gifts from Dr. Hosrt Zahner (Institute of Parasitology, Justus-Liebig-University Giessen, Giessen, Germany) and Dr. Ronald Wenger (Institute of Physiology, University of Zürich-Irchel, Zürich, Switzerland) originally synthetized by Novartis (Basel, Switzerland). Of all non-immunosupressive CsA derivatives tested, H-7-94 and F-7-62 showed the best parasiticidal effects in micromolar levels against the three forms of the parasite life-cycle, and had non-toxic effects for mammalian cells [36,40].

In an in vivo experimental approach, mice were treated with H-7-94 and F-7-62 CsA analogs before and during the first five days post-infection with *T. cruzi* bloodstream forms. Drug treated mice survived (100%) after parasite challenge and had significant lower parasitemia than non-treated mice control (60% survival) [36].

Even though the effects of CsA and its derivatives on unicellular parasites of sanitary public heath importance have been evidenced, the current knowledge about the anti-parasitic mechanisms of these drug is rather limited.

## 4. The Effects of Cyclosporin A on *T. cruzi* Cyclophilins

To characterize enzymatic activity from *T. cruzi* cyclophilins, recombinant proteins were obtained. All parasite CyPs proved to have PPIase activity (measured as described in Reference [41]). The enzymatic activity was inhibited by CsA and the non-immunosuppressive derivatives H-7-94 and F-7-62, and showed a positive correlation with anti-parasitic effects [36]. Three-dimensional molecular modelling studies on the structure of *Tc*CyP19 complexed with the most effective CsA analogs suggested which putative protein-ligand binding residues were involved. The simulated interactions with the compounds H-7-94 and F-7-62 showed a total potential complex energy that exhibited correlation with the experimental trypanocidal activity observed [42]. The small and cytosolic cyclophilin from humans (hCyPA), and those from parasitic protozoa as *Plasmodium falciparum* (*Pf*CyP19), *Toxoplasma gondii* (*Tg*CyP18), *Leishmania major* (*Lm*CyP19), and *Trypanosoma cruzi* (*Tc*CyP19) showed a similar CsA inhibition profile of its PPIase activity [36,43,44].

CsA has also been described as an inhibitor of the mitochondrial permeability transition pore (mPTP) in mammals, a pore in the mitochondria which opens under stressful conditions such as Ca^2+^ overload and oxidative stress, allowing the release of apoptotic-related factors into the cytosol [45]. The only component that has been addressed as indispensable for mPTP opening is the Cyclophilin D (CyPD-*Ppif* gene) [46]. CsA binds to CyPD, causing a “desensitization” effect and inhibiting mPTP opening [47].

**Figure 2:**
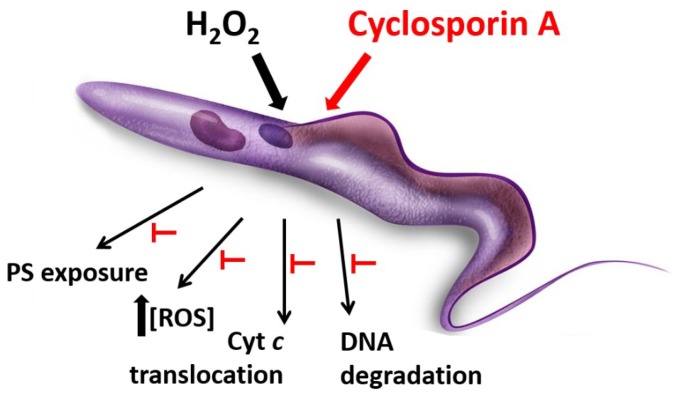
Protective effect of Cyclosporin A against oxidative stress. *T. cruzi* epimastigotes incubated with H_2_O_2_ exhibit cell death features such as mitochondrial membrane depolarization, parasite DNA fragmentation, phosphatidylserine (PS) exposure to the external membrane surface, an increase in reactive oxygen species production and cytochrome c translocation from mitochondrion to cytosol. All these events do not occur when parasites are pre-treated with 1μM Cyclosporin A.

Regarding oxidative stress, CsA specifically inhibited cell death events on *T. cruzi* parasites under hydrogen peroxide stimulus as mitochondrial membrane depolarization, parasite DNA fragmentation, phosphatidylserine exposure to the external membrane surface, and Annexin V binding, reactive oxygen species production and cytochrome *c* translocation from mitochondrion to cytosol (Figure 2) [48]. This suggested that CsA would be inhibiting a regulated cell death process in this protozoan parasite, as occurs in mammalian cells by CsA-CyPD binding.

mPTP opening had not been previously described for a parasitic protozoan and it has been only suggested for *Leishmania* parasites [49]. Based on our previous results on oxidative stress damage, we investigated whether this cell death mechanism through mPTP opening may occur in *T. cruzi*. Our results demonstrated the presence of the mPTP in the parasite. Experiments were performed with a highly selective indicator of mPTP opening as the fluorescent dye calcein-AM co-loaded with CoCl_2_ as a quencher. CsA incubation showed increased calcein retention in the mitochondrion, (60%) and when other mPTP inhibitors (described for mammalian cells) were added, as Bongkrekic acid and ADP, up to 86% calcein retention was achieved, suggesting the existence of a mPTP-like pore in *T. cruzi* parasites, with similar proteins to the mammalian mitochondrial pore, and among them, a mitochondrial cyclophilin orthologue to CyPD [48].

The observation that CsA inhibited some cell death events in *T. cruzi* and other protozoan parasites allowed a better understanding of this pathway. However, further research needs to be done to identify all the effector molecules involved in this process.

## 5. Functional Features of *T. cruzi* Peptidyl-Prolyl *cis-trans* Isomerases

The *Trypanosoma cruzi* life cycle starts in a mammal reservoir and a triatomine bug serves as the vector. In mammals, the bloodstream and infectious forms are called trypomastigotes, and must cross a network of membrane proteins to invade the host cells. The replicative and intracellular form of the parasite is the amastigote, and it can persist for a long time in different organs establishing a chronic infection.

The parasite cyclophilin of 19 kDa has been involved as a key mediator, enhancing parasite survival through the gastrointestinal tract of the reduviid vector. The novel parasite pathway proposed is that the secreted *Tc*CyP19 binds and neutralizes an insect antimicrobial lytic peptide, activating calcineurin intracellular signaling [26]. Besides, the protein expression and mRNA transcription of the *T. cruzi* cyclophilin of 19 kDa were twofold higher in those parasites populations who were in vitro induced to be resistant to the parasiticidal drug benznidazol. The chaperoning activity of the overexpressed *Tc*CyP19 could be associated with the activity of enzymes with antioxidant defense conferring the parasite resistance to benznidazol [50].

**Figure 3:**
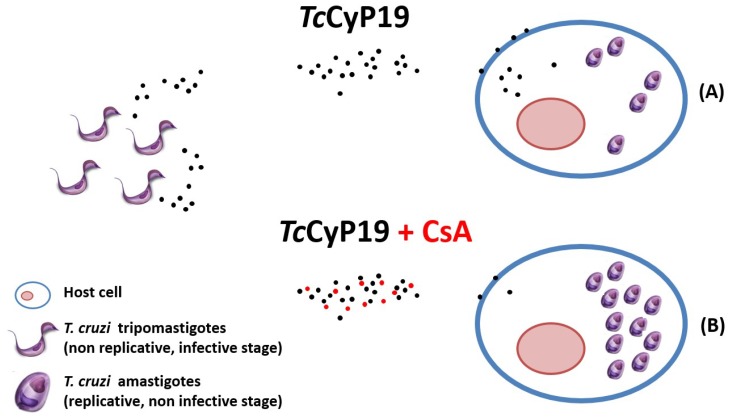
*Tc*CyP19 is abundantly expressed in *T. cruzi*, and interacts with mammalian cells, a process that was partially inhibited by Cyclosporin A (CsA). The pre-incubation of cyclophilin *Tc*CyP19 with VERO cells restrained the parasite penetration (**A**), and again, pre-incubation of *Tc*CyP19 with its inhibitor CsA or specific polyclonal antibodies recovered the percentage of parasite infected cells in control experiments (**B**). The inhibitory penetration effect of *Tc*CyP19 cyclophilin suggests a protective role of this protein to assure the survival of the infected tissue and allow parasite persistence.

*Tc*CyP19 is abundantly expressed in *T. cruzi*, and interacts with mammalian cells, association that could be confirmed by flow cytometry, and furthermore, this protein was found inside VERO cells, a process that was partially inhibited by CsA. The incubation of cyclophilin *Tc*CyP19 with VERO cells restrained the parasite penetration, and again, pre-incubation of *Tc*CyP19 with its inhibitor CsA or specific polyclonal antibodies recovered the percentage of parasite-infected cells in control experiments. The inhibitory penetration effect of *Tc*CyP19 cyclophilin suggests a protective role of this protein to assure the survival of the infected tissue and allow parasite persistence (Figure 3) (unpublished results). *Tc*CyP19 cyclophilin is suspected to have a very interesting role in the parasite-host relationship, so we intended to study its mammalian target protein using different approaches as pull down, immunoprecipitation, and protein crosslinkers, but did not find any substrates in these assays, suggesting a transient protein-protein interaction, as it has also been observed for *Mycobacterium tuberculosis* [51].

Regarding the CsA cytoprotective effects observed in *T. cruzi* that undergo oxidative stress, we proposed the existence of a mitochondrial transition pore-like in the parasite, and searched for an homologue of the mammalian CyPD in the parasite as target. *Tc*CyP22 protein, which could be isolated by CsA affinity chromatography [17], showed a putative mitochondrial localization, and its localization to mitochondria was confirmed in the three stages of the parasite life cycle [52]. Oxidative stress was induced in overexpressing *Tc*CyP22 parasites and an enhanced loss of mitochondrial membrane potential and cell viability were significantly different from control parasites, defining an homologue of CyPD for the first time in a protozoan parasite, and the involvement of this protein in *T. cruzi* regulated cell death [52,53].

Other parasitic protozoan small cyclophilins have been involved in several cell functions as *L. braziliensis* and *L. infantum* resistance to anti-parasitic drugs [54], aggregation-disaggregation of proteins in *Leishmania donovani* [55,56], and the interesting fact that *Toxoplasma gondii Tg*CyP18 binds the CCR5 receptor blocking HIV infection on human T cells [57]. Regarding larger protozoan cyclophilins, mutants of *L. donovani* CyP40 showed a defect in its virulence, as parasites fail to establish an intracellular infection [58], and upregulates several stress proteins, probably to compensate for the lack of the CyP40 [59].

## 6. Concluding Remarks

Though molecular aspects have been extensively described for cyclophilins in diverse parasites affecting humans such as *Plasmodium falciparum* [43,60], *Toxoplasma gondii* [44,61], *Leishmania major* [62], *L. donovani* [63], *Trypanosoma brucei* [24] and *T. cruzi* [17], the functional role of many expressed parasitic unicellular cyclophilins remains unknown, requiring further investigation.

The final assessment of the biological functions of *T. cruzi* cyclophilins will require successful gene knock out and overexpression, currently ongoing.
